# ReGaTE: Registration of Galaxy Tools in Elixir

**DOI:** 10.1093/gigascience/gix022

**Published:** 2017-04-10

**Authors:** Olivia Doppelt-Azeroual, Fabien Mareuil, Eric Deveaud, Matúš Kalaš, Nicola Soranzo, Marius van den Beek, Björn Grüning, Jon Ison, Hervé Ménager

**Affiliations:** 1 Centre de Bioinformatique, Biostatistique et Biologie Intégrative (C3BI, USR 3756 Institut Pasteur et CNRS), 25 rue du Docteur Roux, Paris, France; 2 Computational Biology Unit, Department of Informatics, University of Bergen, Thormøhlensgate 55, Bergen, Norway; 3 Earlham Institute, Norwich Research Park, NR4 7UG Norwich, United Kingdom; 4 Institut de Biologie Paris-Seine, Université Pierre et Marie Curie, Paris, France; 5 Department of Computer Science, Albert-Ludwigs-University,Center for Biological Systems Analysis (ZBSA), University of Freiburg, Freiburg, Germany; 6 Department of Systems Biology, Center for Biological Sequence Analysis, Technical University of Denmark, Building 208, 2800 Kongens, Lyngby, Denmark.

**Keywords:** Galaxy, bio.tools, bioinformatics services

## Abstract

**Background:** Bioinformaticians routinely use multiple software tools and data sources in their day-to-day work and have been guided in their choices by a number of cataloguing initiatives. The ELIXIR Tools and Data Services Registry (bio.tools) aims to provide a central information point, independent of any specific scientific scope within bioinformatics or technological implementation. Meanwhile, efforts to integrate bioinformatics software in workbench and workflow environments have accelerated to enable the design, automation, and reproducibility of bioinformatics experiments. One such popular environment is the Galaxy framework, with currently more than 80 publicly available Galaxy servers around the world. In the context of a generic registry for bioinformatics software, such as bio.tools, Galaxy instances constitute a major source of valuable content. Yet there has been, to date, no convenient mechanism to register such services en masse. **Findings:** We present ReGaTE (Registration of Galaxy Tools in Elixir), a software utility that automates the process of registering the services available in a Galaxy instance. This utility uses the BioBlend application program interface to extract service metadata from a Galaxy server, enhance the metadata with the scientific information required by bio.tools, and push it to the registry. **Conclusions:** ReGaTE provides a fast and convenient way to publish Galaxy services in bio.tools. By doing so, service providers may increase the visibility of their services while enriching the software discovery function that bio.tools provides for its users. The source code of ReGaTE is freely available on Github at https://github.com/C3BI-pasteur-fr/ReGaTE.

## Introduction

Over the recent years, various initiatives have aimed at cataloguing bioinformatics tools and services [1–6]. In particular, the ELIXIR Tools and Data Services Registry (bio.tools) [7] offers a community-curated information portal whose goals are comprehensive coverage and consistent description of bioinformatics tools and services. Another ongoing trend is the integration of bioinformatics software in workbench and workflow environments, which allows data analysts to design, automate, and reproduce bioinformatics experiments.

The Galaxy framework [8–10] is one of the most popular of such environments, with currently more than 80 publicly available Galaxy servers (see https://wiki.galaxyproject.org/PublicGalaxyServers) around the world. The registration and maintenance of entries in the bio.tools registry is based on a “federated curation model” whereby the maintenance of resource entries is handled by their owners. Current efforts to automatically register tools and services in resource catalogs mostly target programming language–specific catalogs, such as the Python package index (see http://pypi.python.org/pypi), and seldom domain-specific catalogs, with a few notable exceptions such as BioJS [11], the BioGems registry [12], and BioMOBY [13]. In contrast, ReGaTE is a solution for owners of Galaxy server instances to easily register their tools on a resource catalog that is not specific to any programming language or technical requirement. The scientists browsing the bio.tools server can therefore search and compare resources independently of any technological implementation. In the context of a generic registry for bioinformatics software, such as bio.tools, Galaxy instances constitute a major source of valuable content. The ReGaTE utility is a software component that automates the registration of the bioinformatics tools installed on a Galaxy server. We will present in the following sections the major aspects of its implementation, its architecture, and finally the mapping of tool metadata from Galaxy to bio.tools.

## Implementation

ReGaTE pulls tool descriptions from a Galaxy server, augments the information, and pushes it to the bio.tools registry.

A **Galaxy server** is a framework that supports users to configure and run a range of bioinformatics tools and workflows and that gathers many other features for the sharing, visualization, and reproducibility of analyses. The user interface and execution of tools are based on a tool definition in an eXtensible Markup Language (XML) file (detailed documentation of this format is available at https://wiki.galaxyproject.org/Admin/Tools/ToolConfigSyntax). Each file describes the bioinformatics tool in a detailed way, including the tool parameters, inputs, and outputs. This allows the display of their sometimes complex configuration options in a graphical user interface, primarily to enable tool parameterization and its execution. Such tool definitions are loaded by the Galaxy server and are accessible through the Galaxy RESTful interface. The BioBlend library [14] allows convenient access to the Galaxy application program interface (API) from Python. Here, we have used BioBlend to extract Galaxy tool definitions from remote Galaxy instances.

**Bio.tools** [7] is a web portal provided by ELIXIR – the European infrastructure for biological information for the exploration of bioinformatics resources including software packages, web services, and database portals. Through a dedicated graphical interface, users can search for and compare resources. Thus, bioinformatics resource providers can use bio.tools to enhance the visibility of their services. The description and registration of a resource can be done manually via a web user interface, or resources may be registered using the registry API. Registry entries follow a model that is formalized in biotoolsSchema (the biotoolsSchema format definition is available at https://github.com/bio-tools/biotoolsSchema/), an XML schema that defines a resource description model for bioinformatics with a mandatory core of 10 attributes.

**ReGaTE** fetches the Galaxy tool definitions, enhances them with additional annotations, and converts them into the biotoolsSchema-based JSON format, using the mapping mechanism described in the next section, before pushing them to bio.tools. This process can be triggered all at once or step by step, first extracting the tool metadata, and second pushing enhanced metadata to bio.tools. A ReGaTE user needs to have an account on the targeted Galaxy and retrieve his API key to extract the tool definitions and an account on the bio.tools server to push the registry entries.

## ReGaTE Architecture

**Figure 1:**
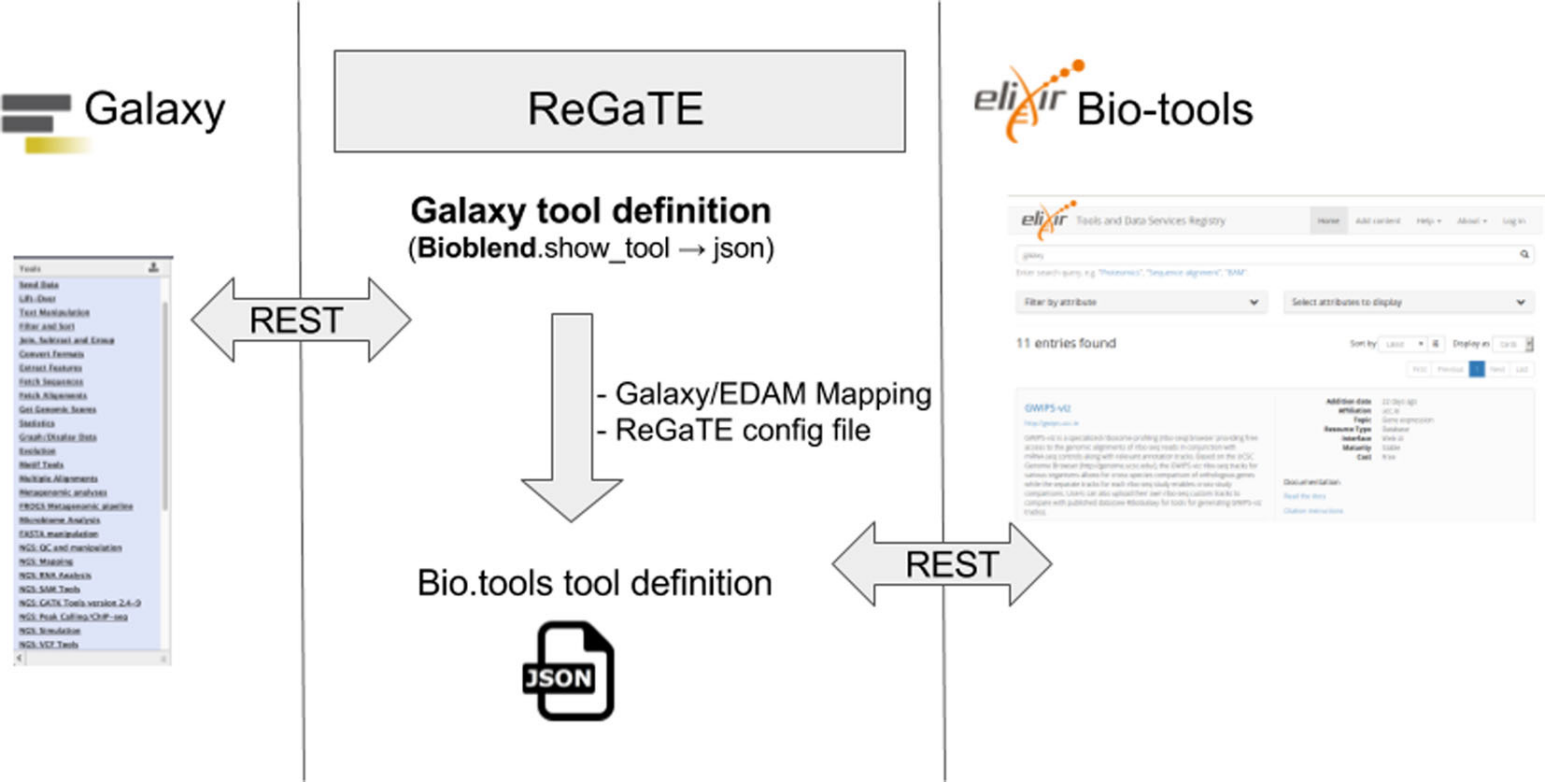
ReGaTE software architecture.

ReGaTE is a Python script coupled with a configuration file and mapping of semantics used by Galaxy and bio.tools. An overview of its architecture is shown in Fig. 1.

The configuration file includes the Galaxy server uniform resource locator, an API key, and a directory to store the generated tool files uploadable to bio.tools. Suffix and prefix variables, for tagging the names of the tools extracted by ReGaTE, may also be specified. For example, the name of the tool SARTools DESeq2 [15], implemented at Institut Pasteur, can be named SARTools DESeq2-IP.

## Tool Metadata Mapping

A biotools-Schema file describes a given software application, covering different properties:

- scientific properties, such as the domain catered for and description of the type of task(s) done by a tool;
- technical properties, such as the type of software and its interface(s), e.g., command line tool, web application, web service, etc.;
- credit, e.g., the references that need to be cited when referring to this work;
- administrative information, such as the license used in the software.

Some of these properties are described using the EMBRACE Data and Methods (EDAM) ontology [16]. Development of the EDAM ontology is driven by community requests via GitHub, mailing lists, and community-based hackathons (more information on contributions can be found at https://github.com/edamontology/edamontology/blob/master/HOW_TO_CONTRIBUTE.md). It currently includes 3280 concepts with regular (at least quarterly) major releases. EDAM includes the following common bioinformatics concepts:

- **topics**, i.e., scientific disciplines or domains covered by the resource;
- specific **operations** performed by a tool or service;
- **types** of input and output data;
- **formats** in which inputs and outputs are available.

The mapping from a Galaxy tool definition file (detailed documentation of this format is available at https://wiki.galaxyproject.org/Admin/Tools/ToolConfigSyntax) to a bio.tools file is handled by the ReGaTE code, taking advantage of the important number of common properties between such workbench wrappers and registry entries [17]. A few properties are not natively available in the Galaxy tool files retrieved by BioBlend; these missing data are provided by the ReGaTE configuration files.

The mapping of Galaxy tool properties to EDAM concepts is a key component. This translation is handled by yet another markup language mapping files included in the ReGaTE distribution that handle the conversion of Galaxy datatypes to EDAM data and format concepts, and that also allow EDAM topics and operations to be specified.

### Conclusions and Future Work

The bio.tools registry allows Galaxy server maintainers to increase the visibility of their services, set in context of offerings from other providers. The ReGaTE utility is a fast and convenient solution to enhance, publish, and maintain the services provided by a Galaxy server in the registry. Furthermore, ReGaTE can prove a valuable contribution toward providing bio.tools with more comprehensive coverage of the community resources.

Current work on ReGaTE is focused on migration of the core functionality and tool semantics to the Galaxy Project itself. This integration will rely on the direct annotation of Galaxy datatypes with EDAM format and data concepts (see https://github.com/galaxyproject/galaxy/pull/2387 and https://github.com/galaxyproject/galaxy/pull/2428), as well as the possibility to specify an EDAM topic (see https://github.com/galaxyproject/galaxy/pull/2397) and operational concepts (see https://github.com/galaxyproject/galaxy/pull/2379) directly in Galaxy tool definitions (see https://github.com/galaxyproject/galaxy/pull/3221).

The use of EDAM as a standard for describing bioinformatics resources can provide a backbone to improve interoperability and guide users to connect and compose Galaxy tools [18], extending potentially to external components and environments that share this common vocabulary. A future priority will therefore be to exploit EDAM annotations in these ways for the benefit of Galaxy users and providers.

## Abbreviations

API: application program interface; EDAM: EMBRACE Data and Methods; URL: Uniform Resource Locator; XML: eXtensible Markup Language; YAML: Yet Another Markup Language.

## Supplementary Material

GIGA-D-16-00051_Original_Submission.pdf

GIGA-D-16-00051_Revision_1.pdf

GIGA-D-16-00051_Revision_2.pdf

GIGA-D-16-00051_Revision_3.pdf

Response_to_reviewers_comments_Original_Submission.pdf

Response_to_reviewers_Revision_1.pdf

Response_to_reviewer_comments_Revision_2.pdf

Reviewer_1_Report_(Original_Submission).pdf

Reviewer_2_Report_(Original_Submission).pdf

Reviewer_3_Report_(Original_Submission).pdf

Reviewer_3_Report_(Revision_1).pdf

Reviewer_3_Report_(Revision_2).pdf

## Availability and requirements

Project name: ReGaTE

Project home page: https://github.com/C3BI-pasteur-fr/ReGaTE

Operating system(s): Unix-based operating systems

Programming language: Python

License: GPLv2

Any restrictions to use by non-academics: None

## Availability of supporting data

A snapshot of the version of the ReGaTE source code used in this paper is archived in the GigaScience database GigaDB [19].
