# Supplementary material for: ReGaTE: Registration of Galaxy Tools in Elixir
Source: Gigascience. 2017 Apr 10;6(6):1–4. doi: 10.1093/gigascience/gix022 (PMC5530318; doi:10.1093/gigascience/gix022)

Manuscript Click here to download Manuscript regate-registration-galaxy.pdf



RESEARCH

# ReGaTE, Registration of Galaxy Tools in Elixir

Olivia Doppelt-Azeroual[1*†], Fabien Mareuil[1†], Eric Deveaud[1], Matúš Kalaš[2], Nicola Soranzo[3], Marius van den Beek[4], Bjoern Gruening[5], Jon Ison[6] and Hervé Ménager[1]

**Abstract**

Background
Bioinformaticians routinely use multiple software tools and data sources in their day-to-day work, and have been guided in their choices by a number of cataloguing initiatives. The ELIXIR Tools and Data Services Registry (*bio.tools*) aims to provide a central information point, independent of any specific scientific scope within bioinformatics or technological implementation. Meanwhile, efforts to integrate bioinformatics software in workbench and workflow environments have accelerated, to enable the design, automation, and reproducibility of bioinformatics experiments. One such popular environment is the Galaxy framework, with currently more than 80 publicly available Galaxy servers around the world. In the context of a generic registry for bioinformatics software, such as *bio.tools*, Galaxy instances constitute a major source of valuable content. Yet there has been, to date, no convenient mechanism to register such services *en masse*.

Findings
We present ReGaTE (Registration of Galaxy Tools in Elixir), a software utility that automates the process of registering the services available in a Galaxy instance. This utility uses the BioBlend API to extract service metadata from a Galaxy server, enhance the metadata with the scientific information required by *bio.tools*, and push it to the registry.

Conclusion
ReGaTE provides a fast and convenient way to publish Galaxy services in *bio.tools*. By doing so, service providers may increase the visibility of their services, whilst enriching the software discovery function that *bio.tools* provides for its users. The source code of ReGaTE is freely available on Github, at https://github.com/C3BI-pasteur-fr/ReGaTE.

**Keywords:** Galaxy; Bio.Tools; bioinformatics services

## Introduction

Over the recent years, various initiatives have aimed at cataloguing bioinformatics tools and services[1–6]. In particular, the ELIXIR Tools and Data Services Registry[7] (*bio.tools*) offers a community-curated information portal whose goals are comprehensive coverage and consistent description of bioinformatics tools and services. Another ongoing trend is the integration of bioinformatics software in workbench and workflow environments, which allows data analysts to design, automate, and reproduce bioinformatics experiments.

*Correspondence: olivia.doppelt@pasteur.fr
[1]Centre de Bioinformatique, Biostatistique et Biologie Intégrative (C3BI, USR 3756 Institut Pasteur et CNRS) – Paris, France , 25 rue du Docteur Roux, Paris, France
Full list of author information is available at the end of the article
†Equal contributor

The Galaxy framework[8–10] is one of the most popular of such environments, with currently more than 80 publicly available Galaxy servers[1] around the world. The registration and maintenance of entries in the *bio.tools* registry is based on a ‘federated curation model’ whereby the maintenance of resource entries is handled by their owners. Current efforts to automatically register tools and services in resource catalogs mostly target programming-language specific catalogs, such as the Python package index[2], and seldom domain-specific, with a few notable exceptions such as BioJS[11], the BioGems registry[12], or BioMOBY[13]. In contrast, ReGaTE is a solution for owners of Galaxy server instances to easily register their tools on a resource catalog which is not specific of any programming language or technical requirement. The scientists

browsing the *bio.tools* server can therefore search and compare resources independently of any technological implementation. In the context of a generic registry for bioinformatics software, such as *bio.tools*, Galaxy instances constitute a major source of valuable content. The ReGaTE utility is a software component that automates the registration of the bioinformatics tools installed on a Galaxy server. We will present in the following sections the major aspects of its implementation, its architecture, and finally the mapping of tool metadata from Galaxy to *bio.tools*.

## Implementation

ReGaTE pulls tool descriptions from a Galaxy server, augments the information and pushes it to the *bio.tools* registry.

A **Galaxy server** is a framework that supports users to configure and run a range of bioinformatics tools and workflows, and which gathers many other features for the sharing, visualization, and reproducibility of analyses. The user interface and execution of tools are based on a tool definition in an XML file[3]. Each file describes the bioinformatics tool in a detailed way, including the tool parameters, inputs and outputs. This allows to display their sometimes complex configuration options in a graphical user interface, primarily, to enable tool parameterisation and its execution. Such tool definitions are loaded by the Galaxy server, and are accessible through the Galaxy RESTful interface. The BioBlend library[14] allows convenient access to the Galaxy API from Python. Here, we have used BioBlend to extract Galaxy tool definitions from remote Galaxy instances.

**bio.tools**[7] is a web portal provided by ELIXIR - the European infrastructure for biological information - for the exploration of bioinformatics resources including software packages, Web services and database portals. Through a dedicated graphical interface, users can search for and compare resources. Thus, bioinformatics resource providers can use *bio.tools* to enhance the visibility of their services. The description and registration of a resource can be done manually via a Web user interface, or resources may be registered using the registry API. Registry entries follow a model which is formalized in biotoolsSchema[4], an XML schema which defines a resource description model for bioinformatics with a mandatory core of ten attributes.

**ReGaTE** fetches the Galaxy tool definitions, enhances them with additional annotations, and converts them into the biotoolsSchema-based JSON format, using on the mapping mechanism described in the next section, before pushing them to *bio.tools*. This process can be triggered all at once or step by step, first extracting the tool metadata, and second pushing enhanced metadata to *bio.tools*. A ReGaTE user needs to have an account on the targeted Galaxy and retrieve his API key to extract the tool definitions, and an account on the *bio.tools* server to push the registry entries.

## ReGaTE architecture

ReGaTE is a Python script coupled with a configuration file and mapping of semantics used by Galaxy and *bio.tools*. An overview of its architecture is shown in (Figure 1).

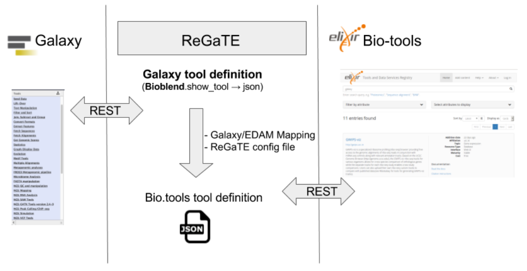


**Figure 1** ReGaTE software architecture

The configuration file includes the Galaxy server URL, an API key, and a directory to store the generated tool files uploadable to *bio.tools*. Suffix and prefix variables, for tagging the names of the tools extracted by ReGaTE, may also be specified. For example, the name of the tool `sartools_deseq2`[15], implemented at Institut Pasteur can be named `sartools_deseq2-IP`.

## Tool metadata mapping

A biotoolsSchema file describes a given software application, covering different properties:

- scientific properties, such as the domain catered for and description of the type of task(s) done by a tool.
- technical properties, such as the type of software and its interface(s), e.g., command line tool, Web application, Web service etc.
- credit, for instance the references that need to be cited when referring to this work
- administrative information, such as the license used in the software

Some of these properties are described using the EDAM ontology[16]. Development of the EDAM ontology is driven by community requests via GitHub, mailing lists and community-based hackathons[5]. It currently includes 3280 concepts with regular (at least

quarterly) major releases. EDAM includes the following common bioinformatics concepts:

- **topics** i.e., scientific disciplines or domains covered by the resource
- specific **operations** performed by a tool or service
- **types** of input and output data
- **formats** in which inputs and outputs are available

The mapping from a Galaxy tool definition file[3] to a *bio.tools* file is handled by the ReGaTE code, taking advantage of the important number of common properties between such workbench wrappers and registry entries [17]. A few properties are not natively available in the Galaxy tool files retrieved by BioBlend; these missing data are provided by the ReGaTE configuration files.

The mapping of Galaxy tool properties to EDAM concepts is a key component. This translation is handled by YAML mapping files included in the ReGaTE distribution that handle the conversion of Galaxy datatypes to EDAM data and format concepts, and which also allow EDAM topics and operations to be specified.

## Conclusion and future work

The *bio.tools* registry allows Galaxy server maintainers to increase the visibility of their services, set in context of offerings from other providers. The ReGaTE utility is a fast and convenient solution to enhance, publish and maintain the services provided by a Galaxy server in the registry. Furthermore, ReGaTE can prove a valuable contribution toward providing *bio.tools* with more comprehensive coverage of the community resources.

Current work on ReGaTE is focused on migration of the core functionality and tool semantics to the Galaxy Project itself. This integration will rely on the direct annotation of Galaxy datatypes with EDAM format and data concepts[6], as well as the possibility to specify EDAM topic[7] and operation[8] concepts directly in Galaxy tool definitions[9].

The use of EDAM as a standard for describing bioinformatics resources can provide a backbone to improve interoperability, and guide users to connect and compose Galaxy tools [18], extending potentially to external components and environments that share this common vocabulary. A future priority will therefore be to exploit EDAM annotations in these ways, for the benefit of Galaxy users and providers.

## Abbreviations

**API**: Application Program Interface
**XML**: eXtensible Markup Language
**URL**: Uniform Resource Locator
**EDAM**: EMBRACE Data and Methods
**YAML**: Yet Another Markup Language

## Availability and requirements

Project name:
ReGaTE

Project home page:
https://github.com/C3BI-pasteur-fr/ReGaTE

Operating system(s):
Unix-based operating systems

Programming language:
Python

License:
GPLv2

Any restrictions to use by non-academics:
None

## Availability of supporting data

A snapshot of the version of the ReGaTE source code used in this paper is archived in the GigaScience database GigaDB [19].

#### Competing interests

The authors declare that they have no competing interests.

#### Author's contributions

ReGaTE was designed by contributions of all authors. The development was done by OD, FM and HM. The paper was written by all of the authors, who read and approved the final manuscript.

#### Acknowledgements

ELIXIR-EXCELERATE is funded by the European Commission within the Research Infrastructures programme of Horizon 2020 [676559]. The authors wish to thank the developers of the *bio.tools* registry, especially Emil Rydza and Piotr Chmura, for the technical support they provided during the development of this tool. They also thank Bertrand Néron for the contribution of his technical expertise in Python.

#### Notes

[1] see https://wiki.galaxyproject.org/PublicGalaxyServers
[2] See http://pypi.python.org/pypi
[3] Detailed documentation of this format is available at https://wiki.galaxyproject.org/Admin/Tools/ToolConfigSyntax
[4] The biotoolsSchema format definition is available at https://github.com/bio-tools/biotoolsSchema/.
[5] More information on contributions can be found at https://github.com/edamontology/edamontology/blob/master/HOW_TO_CONTRIBUTE.md
[6] see https://github.com/galaxyproject/galaxy/pull/2387 and https://github.com/galaxyproject/galaxy/pull/2428
[7] see https://github.com/galaxyproject/galaxy/pull/2397
[8] see https://github.com/galaxyproject/galaxy/pull/2379
[9] see https://github.com/galaxyproject/galaxy/pull/3221

**Author details**
[1]Centre de Bioinformatique, Biostatistique et Biologie Intégrative (C3BI, USR 3756 Institut Pasteur et CNRS) – Paris, France , 25 rue du Docteur Roux, Paris, France. [2]Computational Biology Unit, Department of Informatics, University of Bergen, Thormøhlensgate 55, Bergen, Norway. [3]Earlham Institute, Norwich Research Park, NR4 7UG Norwich, United Kingdom. [4]Institut de Biologie Paris-Seine, Université Pierre et Marie Curie, Paris, France. [5]Department of Computer Science, Albert-Ludwigs-University,Center for Biological Systems Analysis (ZBSA), University of Freiburg, , , Freiburg, Germany. [6]Department of Systems Biology,Center for Biological Sequence Analysis, Technical University of Denmark, Building 208, 2800 Kongens, , Lyngby, Denmark.

**References**

1. Artimo, P., Jonnalagedda, M., Arnold, K., Baratin, D., Csardi, G., De Castro, E., Duvaud, S., Flegel, V., Fortier, A., Gasteiger, E., et al.: Expasy: Sib bioinformatics resource portal. Nucleic Acids Res., 400 (2012)
2. Li, J.-W., Robison, K., Martin, M., Sjödin, A., Usadel, B., Young, M., Olivares, E.C., Bolser, D.M.: The SEQanswers wiki: a wiki database of tools for high-throughput sequencing analysis. Nucleic Acids Res. **40**(suppl 1,D1), 1313–1317 (2012). doi:10.1093/nar/gkr1058. http://nar.oxfordjournals.org/content/40/D1/D1313.full.pdf+html
3. Fox, J.A., Butland, S.L., McMillan, S., Campbell, G., Ouellette, B.F.F.: The Bioinformatics Links Directory: a Compilation of Molecular Biology Web Servers. Nucleic Acids Res. **33**(suppl 2,W1), 3–24 (2005). doi:10.1093/nar/gki594. http://nar.oxfordjournals.org/content/33/suppl_2/W3.full.pdf+html
4. Galperin, M.Y., Rigden, D.J., Fernández-Suárez, X.M.: The 2015 Nucleic Acids Research Database Issue and Molecular Biology Database Collection. Nucleic Acids Res. **43**(suppl 1,D1), 1–5 (2015). doi:10.1093/nar/gku1241. http://nar.oxfordjournals.org/content/43/D1/D1.full.pdf+html
5. Bhagat, J., Tanoh, F., Nzuobontane, E., Laurent, T., Orlowski, J., Roos, M., Wolstencroft, K., Aleksejevs, S., Stevens, R., Pettifer, S., Lopez, R., Goble, C.A.: BioCatalogue: a universal catalogue of web services for the life sciences. Nucleic Acids Res. **38**(suppl 2,W1), 689–694 (2010). doi:10.1093/nar/gkq394. http://nar.oxfordjournals.org/content/38/suppl_2/W689.full.pdf+html
6. McQuilton, P., Gonzalez-Beltran, A., Rocca-Serra, P., Thurston, M., Lister, A., Maguire, E., Sansone, S.-A.: Biosharing: curated and crowd-sourced metadata standards, databases and data policies in the life sciences. Database **2016** (2016). doi:10.1093/database/baw075. http://database.oxfordjournals.org/content/2016/baw075.full.pdf+html
7. Ison, J., Rapacki, K., Ménager, H., Kalaš, M., Rydza, E., Chmura, P., Anthon, C., Beard, N., Berka, K., Bolser, D., *et al.*: Tools and data services registry: a community effort to document bioinformatics resources. Nucleic acids research **44**(D1), 38–47 (2016)
8. Afgan, E., Baker, D., van den Beek, M., Blankenberg, D., Bouvier, D., Čech, M., Chilton, J., Clements, D., Coraor, N., Eberhard, C., Grüning, B., Guerler, A., Hillman-Jackson, J., Von Kuster, G., Rasche, E., Soranzo, N., Turaga, N., Taylor, J., Nekrutenko, A., Goecks, J.: The Galaxy platform for accessible, reproducible and collaborative biomedical analyses: 2016 update. Nucleic Acids Res. **44(W1)**, 3–10 (2016). doi:10.1093/nar/gkw343. http://nar.oxfordjournals.org/content/early/2016/05/02/nar.gkw343.full.pdf+html
9. Goecks, J., Nekrutenko, A., Taylor, J., Team, T.G.: Galaxy: a comprehensive approach for supporting accessible, reproducible, and transparent computational research in the life sciences. Genome Biol. **11**(8), 86 (2010). doi:10.1186/gb-2010-11-8-r86
10. Giardine, B., Riemer, C., Hardison, R.C., Burhans, R., Elnitski, L., Shah, P., Zhang, Y., Blankenberg, D., Albert, I., Taylor, J., Miller, W., Kent, W.J., Nekrutenko, A.: Galaxy: A platform for interactive large-scale genome analysis. Genome Res. **15**(10), 1451–1455 (2005). doi:10.1101/gr.4086505. http://genome.cshlp.org/content/15/10/1451.full.pdf+html
11. Gómez, J., García, L.J., Salazar, G.A., Villaveces, J., Gore, S., García, A., Martín, M.J., Launay, G., Alcántara, R., del-Toro, N., Dumousseau, M., Orchard, S., Velankar, S., Hermjakob, H., Zong, C., Ping, P., Corpas, M., Jiménez, R.C.: BioJS: an open source JavaScript framework for biological data visualization. Bioinformatics **29**(8), 1103–1104 (2013). doi:10.1093/bioinformatics/btt100. http://bioinformatics.oxfordjournals.org/content/29/8/1103.full.pdf+html
12. Bonnal, R.J.P., Aerts, J., Githinji, G., Goto, N., MacLean, D., Miller, C.A., Mishima, H., Pagani, M., Ramirez-Gonzalez, R., Smant, G., Strozzi, F., Syme, R., Vos, R., Wennblom, T.J., Woodcroft, B.J., Katayama, T., Prins, P.: Biogem: an effective tool-based approach for scaling up open source software development in bioinformatics. Bioinformatics **28**(7), 1035–1037 (2012). doi:10.1093/bioinformatics/bts080. http://bioinformatics.oxfordjournals.org/content/28/7/1035.full.pdf+html
13. Wilkinson, M.D., Senger, M., Kawas, E., The BioMoby Consortium: Interoperability with Moby 1.0—It's better than sharing your toothbrush! Brief. Bioinform. **9**(3), 220–231 (2008). doi:10.1093/bib/bbn003. http://bib.oxfordjournals.org/content/9/3/220.full.pdf+html
14. Sloggett, C., Goonasekera, N., Afgan, E.: Bioblend: automating pipeline analyses within galaxy and cloudman. Bioinformatics **29**(13), 1685–1686 (2013)
15. Varet, H., Brillet-Guéguen, L., Coppée, J.-Y., Dillies, M.-A.: Sartools: A deseq2- and edger-based r pipeline for comprehensive differential analysis of rna-seq data. PloS One **11** (2016). doi:e0157022
16. Ison, J., Kalaš, M., Jonassen, I., Bolser, D., Uludag, M., McWilliam, H., Malone, J., Lopez, R., Pettifer, S., Rice, P.: EDAM: An ontology of bioinformatics operations, types of data and identifiers, topics, and formats. Bioinformatics **29**(10), 1325–1332 (2013). doi:10.1093/bioinformatics/btt113. http://bioinformatics.oxfordjournals.org/content/29/10/1325.full.pdf+html
17. Ménager, H., Kalaš, M., Rapacki, K., Ison, J.: Using registries to integrate bioinformatics tools and services into workbench environments. International Journal on Software Tools for Technology Transfer, 1–6 (2015)
18. Lamprecht, A.-L., Naujokat, S., Margaria, T., Steffen, B.: Semantics-based composition of EMBOSS services. J. Biomed. Sem. **2**(Suppl 1), 5 (2011). doi:10.1186/2041-1480-2-S1-S5
19. Doppelt-Azeroual, O., Mareuil, F., Deveaud, E., Kalas, N. M; Soranzo, Van-Den-Beek, M., Gruening, B., Ison, J., Menager, H.: "Supporting Data for "ReGaTE, Registration of Galaxy Tools in Elixir"". doi:10.5524/100287

Figure

Click here to download Figure ReGaTE_figure.png

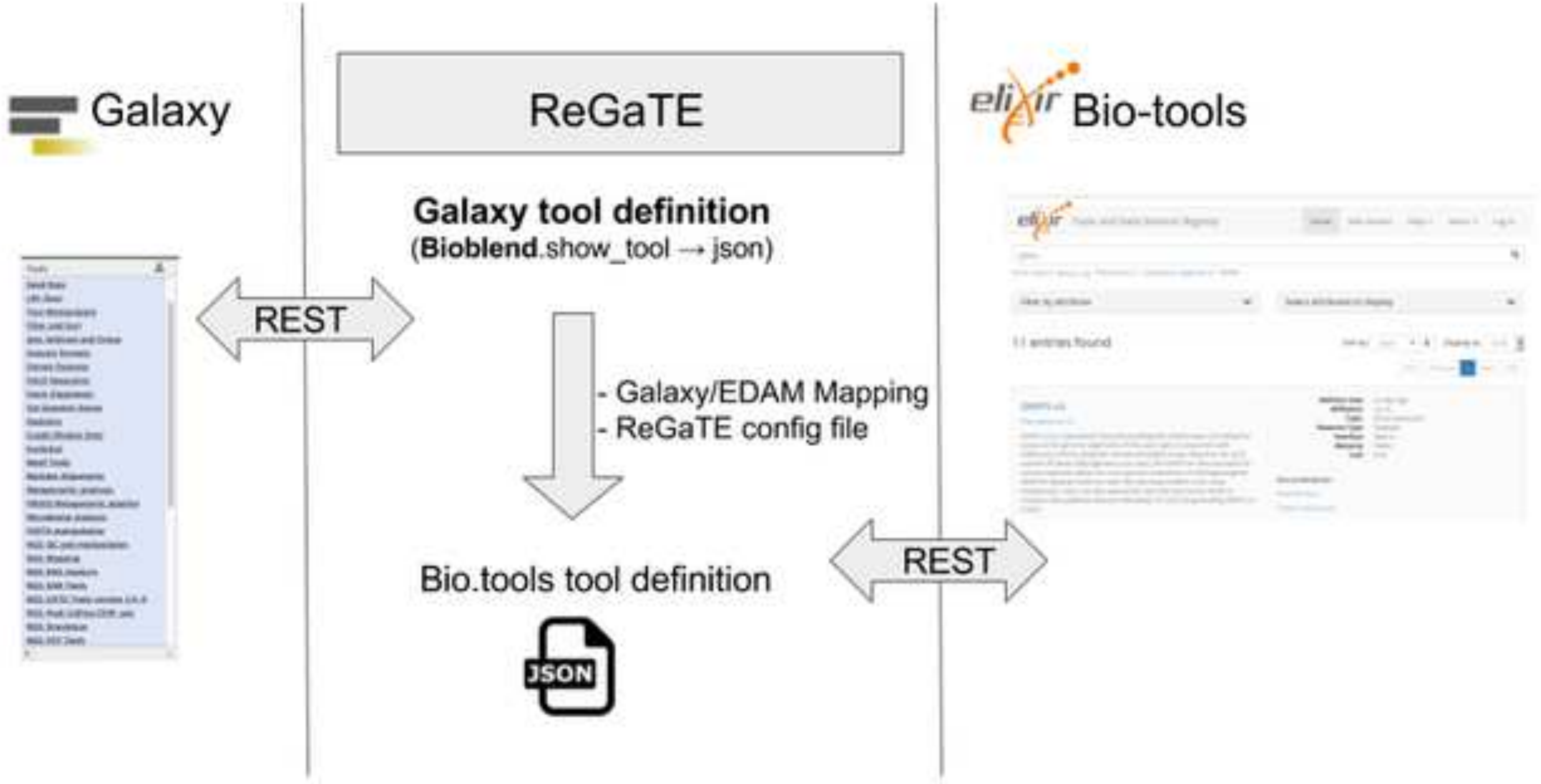

Supplement: GIGA-D-16-00051_Revision_3.pdf [file gix022_GIGA-D-16-00051_Revision_3.pdf]
