# Supplementary material for: ReGaTE: Registration of Galaxy Tools in Elixir
Source: Gigascience. 2017 Apr 10;6(6):1–4. doi: 10.1093/gigascience/gix022 (PMC5530318; doi:10.1093/gigascience/gix022)
Supplement: Response_to_reviewers_comments_Original_Submission.pdf [file gix022_Response_to_reviewers_comments_Original_Submission.pdf]

Olivia Doppelt-Azeroual
Institut Pasteur
25 rue du Dr Roux
75724 Paris
France

To Hans Zauner, GigaScience Editorial Office,
Revised version, GIGA-D-16-00051

Dear Mr Zauner,

We have submitted a revised version of our manuscript entitled: "ReGaTE, Registration of Galaxy Tools in Elixir" by Olivia Doppelt-Azeroual, Fabien Mareuil, et al.

We would like to thank you and the reviewers for reviewing our work. We carefully considered and addressed the reviewers' comments and modified the software and the manuscript accordingly. A reply to the three referees is enclosed and states our arguments concerning each of the reviewer's comments.

I summarize here the main modifications that were made:

The main manuscript modifications were to resolve the errors highlighted by the referees. We also added an abbreviations list like referee #2 suggested, and at last, a few sentences concerning previous similar work were added.
Additionally, corrections were made on the ReGaTE software, according to the remarks raised by the reviewers; moreover we tried to highlight the way to access the documentation which was not clear.

We hope that these modifications and new series of arguments will meet with your approval and that our manuscript will be suitable for publication in GigaScience.

Yours Sincerely,

Olivia Doppelt-Azeroual

Response to reviewer 1

General Comments:

We thank reviewer 1 for this positive feedback.

[REFEREE 1.1]
- P1, L21 - 'provide a central point of information' —> 'provide a central information point'
- P1, L22 - ' independent of any specific scientific scope within bioinformatics or technological implementation' —> independent of any specific scientific scope or technological implementation'
- P1, L28 - 'Yet there has been, to this date; —> 'Yet there has been, to date,'
- P2, L18 - 'allows to display' —> 'allows users to display'
- P2, L22 - 'Restfull interface' —> 'Restful interface'

[ANSWER 1.1] These errors have all been corrected in the manuscript.

[REFEREE 1.2]
- P1, L47 - 'Over the recent years, various initiatives have aimed at cataloguing bioinformatics tools and services[1{3].' there is no mention of initiatives such as the NAR Database issue. Biocatalogue or BioSharing. Shouldn't they be mentioned here?

[ANSWER 1.2]
All of these initiatives are now cited in the manuscript. Additionally, a more extensive list of cataloguing initiatives in a broad sense is available in the cited publication: “Tools and data services registry: a community effort to document bioinformatics resources”.

[REFEREE 1.3] - P2, L45, second column - the EDAM ontology is defined as 'community-defined' - could the authors expand on this? What is the pipeline/timeline for new requests? How many terms does currently EDAM have?

[ANSWER 1.3]
We have modified the manuscript to clarify this point.

Response to reviewer 2

[REFEREE 2.1]
* The manuscript is missing the list of abbreviations.

[ANSWER 2.1]
The manuscript now includes this list.

[REFEREE 2.2]
* The authors mention 80 Galaxy servers available to the public, but the data is not defined as large scale within the context of this field of research. A discussion of the number of tools on each server and the number of tools in common in comparing server tool lists would improve the manuscript.

[ANSWER 2.2]
The referee raises a very interesting point. It is actually not currently straightforward to retrieve programmatically the list of the available tools in all public Galaxy servers. Indeed, the Galaxy API which ReGaTE relies on requires an account on each Galaxy server that is used. Today, more than 80 public Galaxy servers are listed on the Galaxy project webpage (https://wiki.galaxyproject.org/PublicGalaxyServers). Bio.tools project relies on a community effort, based on the fact that the Galaxy administrators should themself extract their tools list in order to enrich bio.tools, and at the same time promote their servers. It is one of the expected outcomes of the bio.tools and ReGaTE projects, to enable any Galaxy administrator to list their tools in a central and publicly available point of access, the bio.tools registry. The bio.tools data will therefore enable the analysis and discussion of the bioinformatics software and services landscape, including Galaxy tools.

[REFEREE 2.3]

* A description of previous work is given only for the registry. Previous work is not described for other efforts to automatically register tools and data services in resource catalogs. Also missing is a discussion of other workbench and workflow environments which were not used. Galaxy is mentioned as the most popular. The manuscript would be of more educational value if additional information was given regarding the choice compared to other environments.

[ANSWER 2.3]
Current efforts to automatically register tools and services in resource catalogs mostly target programming-language specific catalogs, such as the Python package index (https://pypi.python.org/pypi), and seldom domain-specific, with a few notable exceptions such as BioJS (http://www.biojs.io/), BioGems (http://biogems.info/), or PlayMoby. In contrast, ReGaTE is a solution for owners of Galaxy server instances to easily register their tools into a resource catalogue which is not specific to any programming language or technical requirement. The scientists browsing the bio.tools server can therefore search and compare resources independently of any technological implementation. The manuscript now includes these remarks. Regarding the choice of Galaxy, even though the ReGaTE utility is dedicated to Galaxy tools, the HTTP-based API used to automatize the registration of entries in bio.tools is completely provider-agnostic, and it is indeed already used to import entries from other resources, such as SeqWIKI (https://github.com/bio-tools/biotoolsConnect) or the Mobyle workbench (https://github.com/bio-tools/ReMoTE).

[REFEREE 2.4]
* The amount of manual work needed in the automatic registration of the resource is difficult for a casual reader to ascertain from the manuscript. To get a better idea of what is required I went to the github repository. The repository is friendly to python users. The doc folder contained a Makefile for formatting the documentation which required python sphinx. I downloaded the tool on 2 August 2016 to use make.

[ANSWER 2.4]
The ReGaTE documentation is available online at http://regate.readthedocs.io/ and is easily accessible by clicking the docs/latests buttons on the ReGaTe’s GitHub repository. The documentation describes the installation procedure and the execution of the program. There is no need to download and compile the documentation.

[REFEREE 2.5]
* Testing is not fully addressed. It is not discussed in the manuscript at all, and although the software comes with a test directory, it is not documented.

[ANSWER 2.5]
We are sorry if the usage of the test directory was not clear. This directory is available to run some unit tests during development operations but not to realise functional testing. The usage of ReGaTE is explained in the online documentation (there is a link in the GitHub repository).

[REFEREE 2.6]
* The author's python path is hardcoded into the file regate.py.

[ANSWER 2.6]
The source code has been corrected.

[REFEREE 2.7]
* The installation on Mac OS 10.10.5 went smoothly. The software's help command "regate.py -h" did not run successfully: line 79, self. = self.assign("regate_specific_section", "private", ismandatory=True, boolean=True) SyntaxError: invalid syntax

[ANSWER 2.7]
This error has been corrected.

[REFEREE 2.8]
* A possible grammar error is "This allows to display their sometimes complex configuration options in a graphical user interface, primarily, to enable tool parameterisation and its execution." instead of "This allows the display of their sometimes complex configuration options in a graphical user interface, primarily, to enable tool parameterisation and its execution."

[ANSWER 2.8]
The manuscript has been corrected accordingly.

[REFEREE 2.9]
* "Galaxy Restfull interface" should be "Galaxy Restful interface"

[ANSWER 2.9]
The manuscript has been corrected accordingly.

Response to reviewer 3
[REFEREE 3.0]
Unfortunately we were unable to get the source code to run as there are numerous syntax errors in the code that we downloaded. (The git head) There are no installation instructions other than a requirements.txt (listing python deps which we installed). We built and installed the script on a vanilla machine as well as a mac to no avail.

[ANSWER 3.0]
To the best of our knowledge, we have corrected all the remaining errors in the source code. The installation procedure is described in the documentation software available by clicking on the docs/latest buttons in the README of the ReGaTE's GitHub repository.
