# Supplementary material for: ReGaTE: Registration of Galaxy Tools in Elixir
Source: Gigascience. 2017 Apr 10;6(6):1–4. doi: 10.1093/gigascience/gix022 (PMC5530318; doi:10.1093/gigascience/gix022)
Supplement: Response_to_reviewers_Revision_1.pdf [file gix022_Response_to_reviewers_Revision_1.pdf]

Dear Mr. Hans Zauner,
Thank you for your response. Since our last submission, we have improved the installation procedure of ReGaTE, as well as its documentation. Now the installation can be performed with a single command line:
pip install git+https://github.com/C3BI-pasteur-fr/ReGaTE.git
The documentation, available online at http://regate.readthedocs.io, has also been simplified. Don’t hesitate to contact me if you have questions regarding the installation and the use of ReGaTE. We hope these new versions of the program and documentation will clarify the installation procedure for reviewer 3.
The manuscript itself has only been mildly modified, to reflect small modifications in the software architecture, as well as to correct minor typos.
Best wishes to you,
Olivia Doppelt-Azeroual
