# Supplementary material for: ReGaTE: Registration of Galaxy Tools in Elixir
Source: Gigascience. 2017 Apr 10;6(6):1–4. doi: 10.1093/gigascience/gix022 (PMC5530318; doi:10.1093/gigascience/gix022)
Supplement: Response_to_reviewer_comments_Revision_2.pdf [file gix022_Response_to_reviewer_comments_Revision_2.pdf]

Dear Editorial team,

Here is a modified version of the PDF containing the reference to the regate's GigaDB reference.

Best regards,
Olivia
