# Supplementary material for: ReGaTE: Registration of Galaxy Tools in Elixir
Source: Gigascience. 2017 Apr 10;6(6):1–4. doi: 10.1093/gigascience/gix022 (PMC5530318; doi:10.1093/gigascience/gix022)
Supplement: Reviewer_1_Report_(Original_Submission).pdf [file gix022_Reviewer_1_Report_(Original_Submission).pdf]

Reviewer Report

Title: "ReGaTE, Registration of Galaxy Tools in Elixir"

Version: Original Submission **Date:** 8/3/2016

Reviewer name: Pete McQuilton, PhD

Reviewer Comments to Author:

This is a well-researched and well-written manuscript. I commend the authors' commitment to community-driven research and to making their code freely available. I suggest to accept with minor revisions.

Suggested minor revisions:

- P1, L21 - 'provide a central point of information' —> 'provide a central information point'

- P1, L22 - ' independent of any specifc scientifc scope within bioinformatics or technological implementation' —> independent of any specifc scientifc scope or technological implementation'

- P1, L28 - 'Yet there has been, to this date; —> 'Yet there has been, to date,'

- P1, L47 - 'Over the recent years, various initiatives have aimed at cataloguing bioinformatics tools and services[1{3].' there is no mention of initiatives such as the NAR Database issue. Biocatalogue or BioSharing. Shouldn't they be mentioned here?

- P2, L18 - 'allows to display' —> 'allows users to display'

- P2, L22 - 'Restfull interface' —> 'Restful interface'

- P2, L45, second column - the EDAM ontology is defined as 'community-defined' - could the authors expand on this? What is the pipeline/timeline for new requests? How many terms does currently EDAM have?

Level of Interest

Please indicate how interesting you found the manuscript: An article whose findings are important to those with closely related research interests

Quality of Written English

Please indicate the quality of language in the manuscript: Acceptable

Declaration of Competing Interests

Please complete a declaration of competing interests, considering the following questions:

- Have you in the past five years received reimbursements, fees, funding, or salary from an organisation that may in any way gain or lose financially from the publication of this manuscript, either now or in the future?

- Do you hold any stocks or shares in an organisation that may in any way gain or lose financially from the publication of this manuscript, either now or in the future?
- Do you hold or are you currently applying for any patents relating to the content of the manuscript?
- Have you received reimbursements, fees, funding, or salary from an organization that holds or has applied for patents relating to the content of the manuscript?
- Do you have any other financial competing interests?
- Do you have any non-financial competing interests in relation to this paper?

If you can answer no to all of the above, write 'I declare that I have no competing interests' below. If your reply is yes to any, please give details below.

I declare that I have no competing interests.

I agree to the open peer review policy of the journal. I understand that my name will be included on my report to the authors and, if the manuscript is accepted for publication, my named report including any attachments I upload will be posted on the website along with the authors' responses. I agree for my report to be made available under an Open Access Creative Commons CC-BY license (http://creativecommons.org/licenses/by/4.0/). I understand that any comments which I do not wish to be included in my named report can be included as confidential comments to the editors, which will not be published.

I agree to the open peer review policy of the journal
