# Supplementary material for: ReGaTE: Registration of Galaxy Tools in Elixir
Source: Gigascience. 2017 Apr 10;6(6):1–4. doi: 10.1093/gigascience/gix022 (PMC5530318; doi:10.1093/gigascience/gix022)
Supplement: Reviewer_2_Report_(Original_Submission).pdf [file gix022_Reviewer_2_Report_(Original_Submission).pdf]

Reviewer Report

Title: **"ReGaTE, Registration of Galaxy Tools in Elixir"**

Version: Original Submission **Date:** 8/3/2016

Reviewer name: Lynn Young

Reviewer Comments to Author:

Instructions to authors for technical notes

* The manuscript is missing the list of abbreviations.

Rationale

* The rationale is clearly given.

* The authors mention 80 Galaxy servers available to the public, but the data is not defined as large scale within the context of this field of research. A discussion of the number of tools on each server and the number of tools in common in comparing server tool lists would improve the manuscript.

* A description of previous work is given only for the registry. Previous work is not descibed for other efforts to automatically register tools and data services in resource catalogs. Also missing is a discussion of other workbench and workflow environments which were not used. Galaxy is mentioned as the most popular. The manuscript would be of more educational value if additional information was given regarding the choice compared to other environments.

Reproducibility

* The amount of manual work needed in the automatic registration of the resource is difficult for a casual reader to ascertain from the manuscript. To get a better idea of what is required I went to the github repository. The repository is friendly to python users. The doc folder contained a Makefile for formatting the documentation which required python sphinx. I downloaded the tool on 2 August 2016 to use make.

* Testing is not fully addressed. It is not discussed in the manuscirpt at all, and although the software comes with a test directory, it is not documented.

* The author's python path is hardcoded into the file regate.py.

* The installation on Mac OS 10.10.5 went smoothly. The software's help command "regate.py -h" did not run successfully: line 79, self. = self.assign("regate_specific_section", "private", ismandatory=True, boolean=True) SyntaxError: invalid syntax

Writing

* In general the manuscript is well written.

* A possible grammar error is "This allows to display their sometimes complex configuration options in a graphical user interface, primarily, to enable tool parameterisation and its execution." instead of "This allows the display of their sometimes complex configuration options in a graphical user interface, primarily, to enable tool parameterisation and its execution."

* "Galaxy Restfull interface" should be "Galaxy Restful interface"

I enjoyed reading this manuscript, and although the specifics are for a relatively narrow readership, the idea could be of general interest.

Level of Interest

Please indicate how interesting you found the manuscript: An article whose findings are important to those with closely related research interests

Quality of Written English

Please indicate the quality of language in the manuscript: Acceptable

Declaration of Competing Interests

Please complete a declaration of competing interests, considering the following questions:

- Have you in the past five years received reimbursements, fees, funding, or salary from an organisation that may in any way gain or lose financially from the publication of this manuscript, either now or in the future?
- Do you hold any stocks or shares in an organisation that may in any way gain or lose financially from the publication of this manuscript, either now or in the future?
- Do you hold or are you currently applying for any patents relating to the content of the manuscript?
- Have you received reimbursements, fees, funding, or salary from an organization that holds or has applied for patents relating to the content of the manuscript?
- Do you have any other financial competing interests?
- Do you have any non-financial competing interests in relation to this paper?

If you can answer no to all of the above, write 'I declare that I have no competing interests' below. If your reply is yes to any, please give details below.

I declare that I have no competing interests.

I agree to the open peer review policy of the journal. I understand that my name will be included on my report to the authors and, if the manuscript is accepted for publication, my named report including any attachments I upload will be posted on the website along with the authors' responses. I agree for my report to be made available under an Open Access Creative Commons CC-BY license (http://creativecommons.org/licenses/by/4.0/). I understand that any comments which I do not wish to be included in my named report can be included as confidential comments to the editors, which will not be published.

I agree to the open peer review policy of the journal
